# Xeroderma pigmentosum: case report

**DOI:** 10.1590/1984-0462/2023/41/2021390

**Published:** 2023-03-13

**Authors:** Maria Eduarda Coelho Cordeiro, Luisa Corte Real, Andrea Gisele Pereira Simoni

**Affiliations:** a Universidade Federal de Santa Catarina, Araranguá, SC, Brazil.; b Hospital Infantil Joana de Gusmão, Florianópolis, SC, Brazil.

**Keywords:** Xeroderma pigmentosum, Ultraviolet rays, DNA repair-deficiency disorders, Skin neoplasms, Xeroderma pigmentoso, Raios ultravioleta, Distúrbios no reparo do DNA, Neoplasias cutâneas

## Abstract

**Objective:**

The aim of this study was to describe the disease and treatment and to alert health professionals for the identification of signs and symptoms and the need for an early diagnosis in patients with xeroderma pigmentosum (XP).

**Case description:**

An 8-year-old male patient was referred to the Joana de Gusmão Hospital (HIJG) in 2021 for evaluation and specialized care. Previously, the child was followed in his place of origin by oncologic and palliative care, where he was submitted to surgeries and chemotherapy. He was admitted to the HIJG using vismodegib, acitrein, tramadol, and solar protective measures. On physical examination, there were tumors and disseminated macular verrucous and ulcerated lesions. The imaging examination showed solid and expansive lesions on the face, and atelectasis and fibroscarring changes in the lung. The histopathological report proved the existence of melanocanthoma, carcinoma, and pyogenic granuloma. After the evaluation of the case, no surgery, chemotherapy, or radiotherapy was performed. It was decided to maintain the palliative treatment and to continue the use of tramadol for pain, and vismodegib and acitretin were used to control carcinomas and prophylactic measures.

**Comments:**

The XP is a rare disease of autosomal recessive inheritance whose mechanism comes from failure in the DNA repair by exposure to ultraviolet rays, resulting in lesions on the skin and mucous membranes. They start as sunburns and can progress to melanosis, areas with altered pigmentation, premature aging, poikiloderma, and areas of high risk for neoplasms.

## RESUMO

**Objetivo:**

Descrever a patologia e o tratamento realizado e alertar profissionais de saúde sobre os sinais e sintomas e sobre a necessidade de diagnóstico precoce em pacientes com xeroderma pigmentoso (XP).

**Descrição do caso:**

Paciente do sexo masculino, oito anos, foi encaminhado ao Hospital Joana de Gusmão (HIJG) em 2021, com dianóstico de XP, para avaliação e atendimento especializado. Anteriomente, encontrava-se em serviço de oncologia e de cuidados paliativos em sua cidade de origem, mas já realizara cirurgias e quimioterapias previamente. Foi internado no HIJG em uso de vismodegibe, acitretina, tramadol e medidas de proteção solar. Ao exame físico, apresentou lesões maculares, verrucosas, ulceradas e tumores pelo corpo. Os exames de imagem revelaram lesões sólidas e expansivas na face e atelectasias e alterações fibrocicroscópicas no pulmão. O laudo histopatológico comprovou a existência de melanocantoma, carcinoma e granuloma piogênico. Após a avaliação do caso, optou-se por não realizar cirurgias, quimioterapia nem radioterapia. Decidiu-se manter o tratamento paliativo, continuando o uso de tramadol para dor, vismodegibe e acitretina para o controle de carcinomas e profilaxia à exposição ao sol.

**Comentários:**

O XP é uma doença rara de herança autossômica recessiva, cujo mecanismo provém de falha no reparo do DNA pela exposição à luz ultravioleta, resultando em lesões de pele e mucosas. Inicia-se como queimaduras solares e pode avançar para melanoses, áreas com pigmentação alterada, envelhecimento precoce, poiquilodermia e áreas de alto risco neoplásico.

## INTRODUCTION

First described in 1874 by dermatologist Moriz Kaposi, xeroderma pigmentosum (XP) is a rare disease of autosomal recessive inheritance, which affects about one in a million people in the United States and 45 in every million in Japan.^1^ There are still no reliable epidemiological data about the prevalence in Brazil.

As it results from a failure to correct DNA damage caused by exposure to ultraviolet light, there is a formation of multiple skin lesions, especially in sun-exposed areas, and susceptibility to the onset of skin and mucous membrane cancer.^2^

Etiologically, it can be caused by mutations in up to eight genes: seven of them from XPA to XPG (responsible for removing DNA damage by nucleotide excision) and the last gene (XPV) generating the variant subtype of the disease.^3^ As a result, the clinical features vary according to the subtypes of the disease, but skin lesions usually appear early in life and are triggered by extreme sensitivity to light. They start as severe sunburns, even with minimal exposure, and may progress to solar melanomas, followed by areas of hyper- or hypopigmentation, premature aging, poikiloderma, and areas of high risk for neoplasms.^1,3,4^ In about 20% of cases, depending on the mutated gene, there may still be a strong association with progressive neurological diseases. Moreover, patients usually have involvement of other systems, especially ophthalmological symptoms, which may develop progressive blindness.^1^

The diagnosis of XP is based on the clinical manifestations and their evolution, in addition to the biopsy of the lesions that confirms the suspicion and allows the timely removal of the neoformation.^5^ There are still other innovative ultrastructural tests in the literature, but they go beyond the nosocomial reach, being found only in specialized cytogenetic laboratories, mostly.^1^ Once the diagnosis is confirmed, the management is based on containing pre-malignant and malignant lesions by drug and surgical intervention, in addition to preventive measures such as avoiding exposure to ultraviolet rays and using sunscreen.^6–8^

The objective of this study was to describe the disease and its treatment and to alert health professionals in relation to the need for an early diagnosis of patients with XP.

## CASE REPORT

An 8-year-old male patient was referred by the Chapecó primary care to the Joana de Gusmão Hospital (HIJG) on January 27, 2021, for evaluation and specialized care. Being diagnosed with XP, he had skin lesions mainly in exposed areas.

The firstborn in the family, with no history of consanguineousness between the parents, the patient also has first-degree relatives with the disease (a 3-year-old brother and a deceased paternal cousin). In the investigation, a good support network was found, despite the fragile social conditions, with wooden housing and poor basic sanitation.

At 1 year of age, facial macules began to appear. At age 3, he underwent surgery and two cycles of unspecified chemotherapy for a lower lip squamous cell carcinoma with level 1 adenomegaly, performed in Porto Alegre. At age 5, he moved to Chapecó, where he began to be monitored by the local health team, being under the service of oncology and palliative care. There, squamous cell carcinoma was identified in the right eyelid, right malar region, nasal wing, and left eyelid, in addition to a basosquamous carcinoma above the lip. In 2018 and 2019, basal cell carcinoma of the skin and scalp was diagnosed.

On January 27, 2021, he was admitted to the HIJG, already using vismodegib 150 mg/day; acitretin 25 mg/day; tramadol for pain; sunscreen, but with irregular use; and clothes with a UV protection factor, which was reported small for the patient’s size.

**Figure 1.**
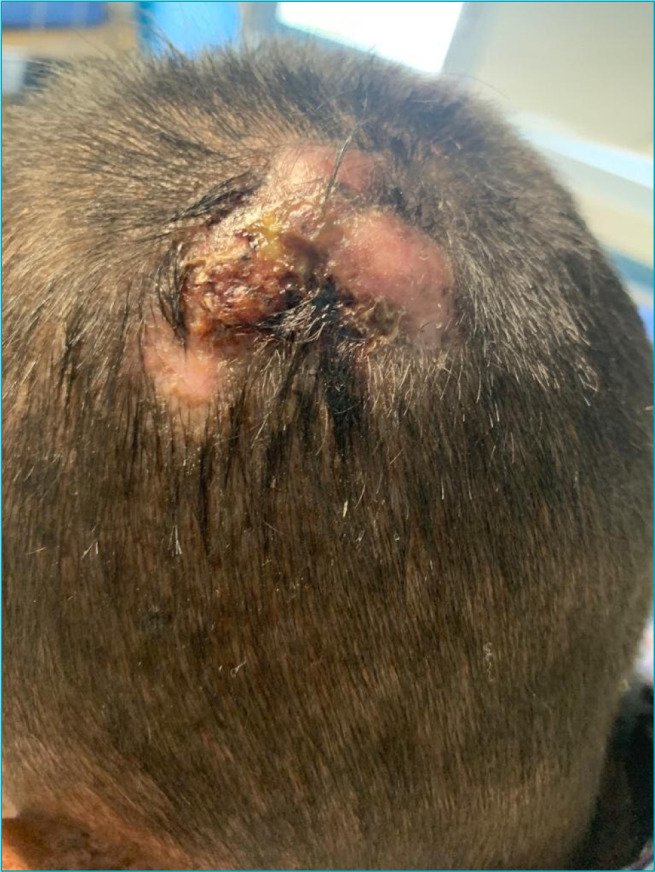
Basal cell carcinoma with ulcerated lesions in scalp measuring about 3 cm in diameter.

**Figure 2.**
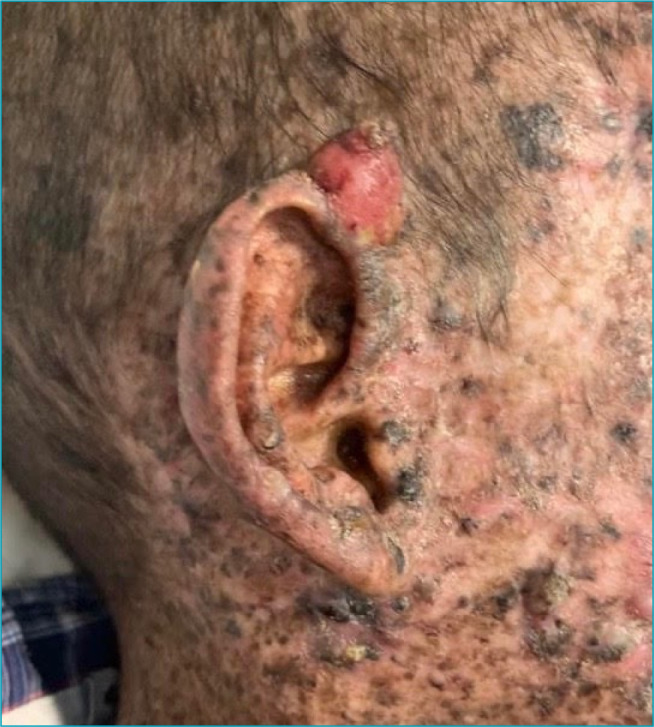
Erythematous tumor with hyperkeratosis at the apex of the right ear measuring about 1.5 cm whose histopathological report failed to differentiate between a basal cell carcinoma or a squamous cell carcinoma.

**Figure 3.**
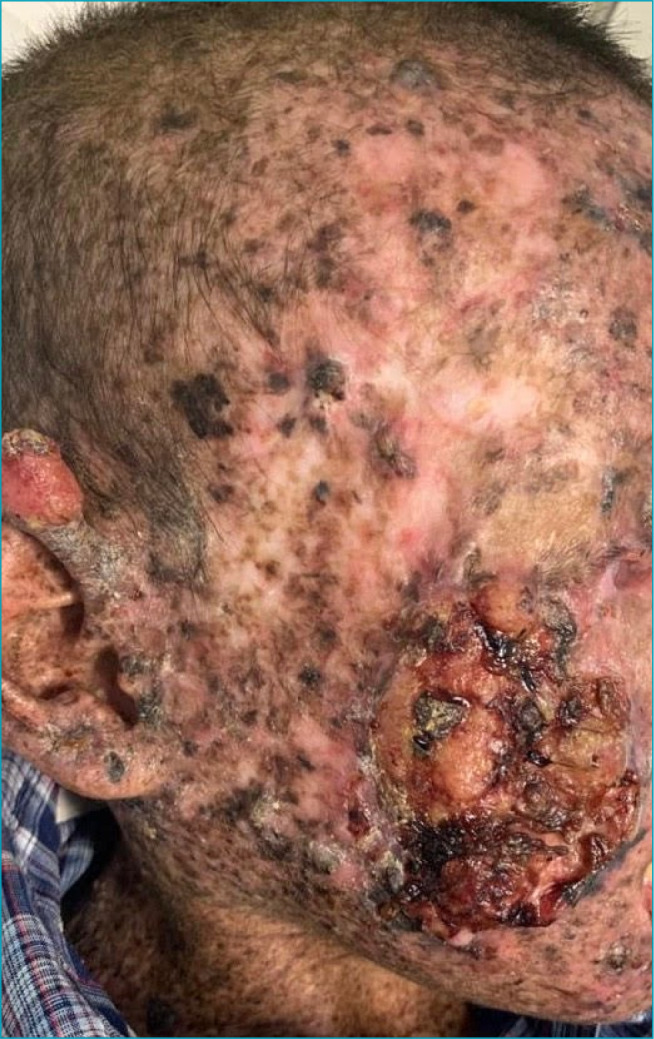
Proliferative epithelial lesion with superficial atypia in the skin of the right temporal region measuring about 7 cm.

**Figure 4.**
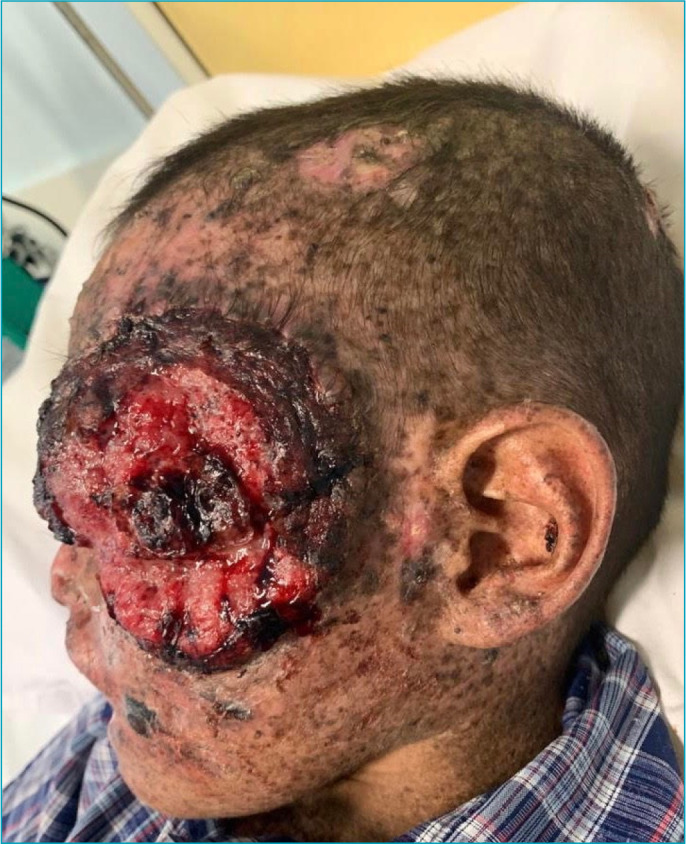
Invasive squamous cell carcinoma in the left orbital lesion of approximately 8 cm in diameter.

On physical examination, macular lesions disseminated throughout the body, less intense in the perineum region, and some verrucous lesions on the face and neck were described. In addition, two ulcerated scalp lesions measuring about 3 cm in diameter were observed (Figure 1); an erythematous tumor with hyperkeratosis at the apex, measuring about 1.5 cm in the helix of the right ear (Figure 2); and a lesion measuring about 7 cm on the right face (Figure 3). Also, blackish lesions were visible on the face and a macerated and bleeding lesion on the tip of the tongue. Finally, the presence of a large lesion in the left orbit was described, measuring approximately 8 cm in diameter (Figure 4), making it impossible to see the eyeball. Due to lesions in the region of the orbit, the patient was already presented with a severe impairment of vision and minimal acuity restricted to the right eye. The remainder of the physical examination was basically unchanged, with auscultation of a 1+/4+ systolic murmur.

The patient was referred for interdisciplinary assessment to a psychologist, speech therapist, dentist, nutritionist, dermatologist, palliative care center, and an oncologist. On the same day of admission, a biopsy of the lesions on the face and a dental evaluation for dental excision were requested. As a care plan, analgesia was performed. A computed tomography scan of the skull, face, chest, and abdomen and a transfusion of filtered and irradiated red cell concentrate were ordered due to anemia. The following day, magnetic resonance imaging (MRI) of the skull and face was also requested, which was scheduled with the patient’s return in February. Given the clinical condition that the management already referred, and the results of the first exams, the patient’s discharge was scheduled on January 29, with regular returns for outpatient follow-up.

The cranial and face tomography result showed that the lesion in the left orbit was invasive, with bone involvement of the lateral wall. Chest tomography showed some supine atelectasis and fibroscarring changes in the anterior segment of the upper lobe of the left lung, and upper abdominal tomography showed no changes. Finally, the MRI report described solid and expansive lesions on both hemifaces, with an aggressive aspect to the left, determining the destruction of the left zygomatic arch and part of the ipsilateral frontal bone.

In the histopathological report, it was possible to prove the existence of a melanocanthoma in the skin biopsy of the cervical region; a carcinoma in the helix of the right ear, whose sample made it impossible to differentiate between basal cell or squamous cell; a proliferative epithelial lesion with superficial atypia in the skin of the right temporal region; an invasive squamous cell carcinoma in the left orbital lesion; and finally a pyogenic granuloma in the oral mucosa of the tongue edge. Immunohistochemistry of the right helix lesion was negative for monoclonal antibodies BER-EP4 and EMA.

After the evaluation of the case, it was decided to maintain the therapeutic approach already carried out during the follow-up of the patient by the Chapecó health team. Thus, given the palliative treatment of this disease, no surgery was performed, not even chemotherapy or radiotherapy, and the use of tramadol for pain, vismodegib and acitretin to control carcinomas, and prophylaxis tools during exposure to the sun were maintained.

## DISCUSSION

Basal cell carcinoma is the most commonly associated carcinoma, followed by squamous cell carcinoma and melanoma, with less common presence of keratoacanthomas, sarcomas, angiomas, and fibromas.^9^ This patient had the three most common types mentioned throughout his evolution and also presented a pyogenic granuloma during follow-up at the service described here. In the literature, the average age described for the appearance of the first nonmelanoma skin cancers in XP is 9 years old,^10^ but the patient reported in this article suffered from the anticipation of the disease, undergoing chemotherapy and surgeries at age 3 due to a squamous cell carcinoma. Similarly, the expected average of melanoma onset would be at 22 years of age;^10^ however, in the case reported, at 8 years of age, one has already been identified in the cervical region.

Moriz Kaposi, in 1874, was the pioneer in describing four patients with “xeroderma,” reporting signs that characterized poikiloderma. Later, pigmented lesions were reported, consolidating the current name of the disease. In 1883, Albert Neisser of Breslau narrated, for the first time, an XP with neurological abnormalities in two brothers. Only in 1968, Cleaver established the molecular origin of XP, when verifying the existence of an abnormality in the processes of DNA repair in fibroblast cultures of patients with the disease irradiated with UV rays.^6^ Since then, the diagnosis of XP has been made through clinical manifestations, its evolution, and histopathological studies of the lesions.^5^

The worldwide incidence of XP is four live births per million inhabitants, although there are geographical variations due to ethnic and genetic reasons. This pathology has an important relationship with the development of melanomas and carcinomas and may increase their incidence up to 2,000 and 10,000 times, respectively. This justifies the importance of early diagnosis and treatment of XP despite its rarity.^10^

In addition to the various skin manifestations, XP also affects other systems: eye problems such as conjunctivitis, xerophthalmia, corneal scarring, and cataracts being extremely common; and neurological problems such as neurodegeneration and brain tumors.^1,3^ Unlike what was observed in the literature, the participant in this article did not have any neurological or ophthalmological dysfunction; however, due to the extensive involvement by infiltrative neoplasms in both orbits, the ophthalmological evaluation was impaired, and it was not possible to draw any exact conclusion. As for neural diseases, physical and imaging examinations made it possible to rule out these affections, with only cranial invasion by facial neoplasms being visualized.

The diagnosis of XP is clinical in addition to biopsies of the lesions that confirm the suspicion of neoplasia and allow the timely removal of the neoformation.^5^ Even today, there are no laboratory or imaging tests to confirm the diagnosis, serving only to control the progress of the disease.^7^ The techniques that allow accurate recognition of the disease remain inaccessible, namely, sequentially genetic and unscheduled DNA synthesis techniques.^1^

Even today, there is no cure for XP, but there are interventions that can be applied at different times in pathological development.^11^ Initially, it was decided not to perform a surgical intervention, as the carcinoma was already locally advanced and there would not be enough healthy skin to perform a graft. Thus, the use of vismodegib was maintained – an oral agent that targets the Hedgehog signaling pathway involved in the development of basal cell carcinoma. This drug was approved after the ERIVANCE study showed a response rate of 45% in people with locally advanced disease and 30% in patients with metastatic basal cell carcinoma after the therapy.^12^ Additionally, acitretin – a synthetic aromatic analog of retinoic acid – was maintained to delay the growth of skin cancer.^13^ Despite the evidence in the literature on the use of topical 5% imiquimod and 5-fluorouracil,^14^ it was decided to keep the abovementioned two medications because they have the same purposes, a good efficacy also in cases of XP and a good adaptation by the patient. Furthermore, as a protective factor, the use of sunscreen was encouraged at times of exposure to ultraviolet radiation, clothing with UV protection and long sleeves, as well as facial protection, as explained in the literature,^15^ however, there was little patient compliance. Also as part of palliative care, tramadol – a weak μ-opioid receptor agonist – was prescribed to alleviate pain in the home environment and morphine during hospitalization.^16^ Finally, a transfusion of irradiated and filtered red blood cell concentrate was performed because of detected anemia.^17^

Despite the rarity of this pathology, XP has a high mortality rate and, therefore, its discussion should become a part of the scientific society. The diagnosis must be early and the follow-up must be continuous and multiprofessional. Although there is still no curative treatment, the use of drugs such as vismodegib and acitretin and sun protection seem to contain the evolution of the disease, in addition to the use of opioids to alleviate pain. Finally, it emphasizes the need for more research on the subject and suggests the development of public strategies aimed at early tracking of XP, considering the epidemiological and hereditary factors involved.
